# Supplementary material for: Exploring the distribution of statistical feature parameters for natural sound textures
Source: PLoS One. 2021 Jun 23;16(6):e0238960. doi: 10.1371/journal.pone.0238960 (PMC8221478; doi:10.1371/journal.pone.0238960)
Supplement: S1 File — (DOCX) [file pone.0238960.s001.docx]

**S1 File.**

**S. Dataset in public repository**

1. All data files (sound textures) used in the study can be accessed at-

<https://figshare.com/articles/media/Natural_Sound_Textures_Files/14229476>

**S.Dataset as zip file.**

2. Interested readers can download all the files in a zip file in the link:

<https://auditoryneuroscience.com/SoundTextures/Natural_Sound_Textures_Files.zip>

Morphed sound can be listened in the following webpage

The morphed sounds from the PC components of Marginals or cochlear correlations or modulation power can be found in our openly accessible webpage in the following link. Interested readers can listen to these morphed sound examples by visiting

<http://auditoryneuroscience.com/Textures/Morphing>
